# Genetic Profiling of MRSA and MSSA from Food Contact Surfaces: Antibiotic, Heavy Metal and Benzalkonium Chloride Resistance

**DOI:** 10.3390/life15121811

**Published:** 2025-11-26

**Authors:** María Guadalupe Avila-Novoa, Oscar Alberto Solis-Velazquez, Jean Pierre González-Gómez, Pedro Javier Guerrero-Medina, Melesio Gutiérrez-Lomelí

**Affiliations:** 1 Centro de Investigación en Biotecnología Microbiana y Alimentaria, Departamento de Ciencias Básicas, División de Desarrollo Biotecnológico, Centro Universitario de la Ciénega, Universidad de Guadalajara, Ocotlan 47820, Jalisco, Mexico; avila.novoa@cuci.udg.mx (M.G.A.-N.); oscar_sv_086@hotmail.com (O.A.S.-V.); 2 Laboratorio Nacional para la Investigación en Inocuidad Alimentaria (LANIIA), Centro de Investigación en Alimentación y Desarrollo, A.C. (CIAD), Culiacan 80110, Sinaloa, Mexico; jean.gonzalez@ciad.mx

**Keywords:** *Staphylococcus aureus*, MRSA, MSSA, food contact surface, multidrug resistant

## Abstract

*Staphylococcus aureus* is a foodborne pathogen that affects animals and humans. The persistence of this pathogen in the environment is associated with its ability to form biofilms and/or develop resistance mechanisms to antibiotics and sanitizers. A total of 67 *S. aureus* strains collected from food contact surfaces (FCSs) made of stainless steel and FCS-polypropylene used in dairy industries in Jalisco, México, were selected for this study. The genetic diversity and genes indicating antibiotic resistance were determined using PCR; antimicrobial susceptibility, resistance to cadmium chloride (CdCl_2_), and the minimum inhibitory concentration (MIC) of benzalkonium chloride (BC) were determined using the agar diffusion method and broth microdilution. Additionally, the effects of BC treatment on biofilm removal were evaluated. A total of 41.7% of the strains were MRSA [SCC*mec* Types II (20.8%), V (13.4%), and IV (7.4%)], and 58.2% were MSSA. Genes encoding antibiotic resistance—*ermC* (2.9%), *ermA* (2.9%), *ermB* (10.4%), *aacA-aphD* (10.4%), *tetM* (17.9%), and *blaZ* (88%)—were detected. A phenotypic test showed that 62.6% of the strains were cadmium-resistant *S. aureus* (>400 µg/mL CdCl_2_), and the MICs of 97% of isolates lay between 1.56 and 25 µg/mL BC. Treatment with BC + MR (100 µg/mL + 1% milk residue*s*) led to a smaller reduction in biofilm (2.11–2.25 log_10_ cfu/cm^2^; *p* < 0.05) compared to BC (3.75–4.03 log_10_ cfu/cm^2^; at 5–10 min). MSSA and MSRA can develop biofilms that harbor mechanism resistance-associated genes, which are a public health hazard and a food safety concern.

## 1. Introduction

*Staphylococcus aureus* represents a significant concern for public health due to its ability to infect both humans and animals, causing a wide spectrum of illnesses such as food poisoning, osteomyelitis, endocarditis, pneumonia, toxic shock syndrome, bloodstream infections, and diverse skin lesions, including abscesses and wound infections [1,2,3]. Additionally, one of the main pathologies associated with *S. aureus* in the agricultural sector is clinical and subclinical mastitis in milk-producing ruminants, generating food safety problems and affecting the commercial value of products such as milk and meat [4,5,6].

*S. aureus* is one of the primary pathogens associated with both clinical and subclinical mastitis in dairy cattle. Comprehensive information on the prevalence and epidemiological status of *S. aureus*–related bovine mastitis in México is still limited and appears to differ among regions, production systems, and herd management practices. Reported rates of subclinical and clinical mastitis range from 39.1 to 67% and 9.3 to 54.5%, respectively, in the states of Guanajuato and Jalisco [7,8]. *S. aureus* can be transmitted to the population by the handling and consumption of contaminated food of animal origin, such as raw milk and dairy products; however, *S. aureus* exhibits diverse resistance mechanisms to various classes of antimicrobials, such as (i) the efflux pump, (ii) immunity and bypassing, (iii) target modification, and (iv) enzyme inactivation [9].

Methicillin-resistant *S. aureus* (MRSA) is a worldwide problem in both healthcare institutions and community settings; moreover, this pathogen has high morbidity and mortality rates and hospital costs [10]. In 2017, the Centers for Disease Control and Prevention (CDC) estimated that MRSA caused 323,700 hospitalizations and 10,600 deaths, with an estimated attributable healthcare cost of USD 1.7B in the United States [11]. According to data from the World Health Organization (WHO), there was a worldwide increase in the proportion of bloodstream infections caused by MRSA from 2016 to 2020, ranging from 20.6% to 32.2% [12].

Moreover, projections indicate that antimicrobial resistance (AMR) could lead to approximately 10 million deaths annually by 2025, with an estimated economic loss of around USD 100 trillion worldwide [13,14]. In the agri-food industry, the continued use of antimicrobials in animal production systems to preserve herd health and productivity has contributed to the emergence of resistant strains [15]. Additionally, compounds such as streptomycin, oxytetracycline, and gentamicin have been applied to control bacterial pathogens in fruit crops including apple, pear, and peach [16].

Furthermore, the generation of waste can negatively impact the diversity of the environmental microbiome, as selection pressure promotes the mobilization and transfer of antibiotic resistance genes (AGRs) to various bacterial species, particularly *S. aureus,* which causes diseases [17,18]. Co-expression of resistance to antibiotics and heavy metals, especially in *S. aureus*, is a public health problem; in addition, this confers resistance to other antibiotics, complicating treatment and posing significant threats to human health and medical practice [10,19].

In the dairy industry, *S. aureus* can form biofilms in food processing environments, such as on food contact surfaces of equipment and/or utensils [20,21]. Biofilms are less susceptible to antimicrobial agents than planktonic cells. In fact, the mechanisms of biofilm resistance and tolerance decrease the effectiveness of antibiotics and disinfectants. For example, (i) the antimicrobial recalcitrance mechanism decreases antibiotic penetration through the biofilm matrix; (ii) β-lactamases secreted into the biofilm matrix inactivate β-lactam antibiotics; (iii) extracellular DNA has high-level binding affinity to vancomycin, which limits its entry into cells embedded in the biofilm matrix; (iv) determinant genes, such as *qac* of *Staphylococcus* spp., encode Qac efflux pumps that pump biocides and disinfectants out of the cell, as well as fluoroquinolones, β-lactams, cetrimide, quaternary ammonium compounds (benzalkonium chloride), and chlorhexidine; and (v) these genes promote the horizontal spread of determinants of antibiotic resistance and complicate the treatment of *S. aureus* infections [22,23,24].

Therefore, the objectives of this research were to determine (i) the prevalence of MSSA and MRSA SCC*mec* Types I–V on food contact surfaces, (ii) the genes associated with the antibiotic and multidrug-resistant (MDR) profiles of MRSA and MSSA on food contact surfaces, (iii) the resistance and/or susceptibility of MRSA and MSSA to benzalkonium chloride (BC) and cadmium chloride (CdCl_2_), and (iv) to evaluate the effects of treatment with BC on the removal of MRSA SCC*me*c Types II, IV, V and MSSA biofilms on stainless steel.

## 2. Materials and Methods

### 2.1. Bacterial Strains

For this research, 67 isolates of *S. aureus* were grown at the Center for Research in Microbial and Food Biotechnology, the Department of Basic Sciences, the Division of Biotechnological Development, the University Center of the Ciénega, the University of Guadalajara. Therefore, this study is retrospective and focused on identifying a comprehensive set of genetic and phenotypic determinants associated with *S. aureus* biofilm formation (Supplementary Table S1). The isolates were obtained from food contact surfaces (FCSs) made of stainless steel and FCS-polypropylene from dairy industries in Jalisco, México [21,25,26].

### 2.2. Detection of Genes Involved in Antibiotic Resistance and Staphylococcal Chromosomal Cassette mec (SCCmec) Typing

The *S. aureus* strains were reactivated in tryptic soy broth (TSB; Becton Dickinson Bioxon, Le Pont de Claix, France) for 24 h at 35 °C. Genomic DNA was extracted using a Bacteria DNA Preparation Kit (Jena Bioscience, Jena, Germany) according to the manufacturer’s instructions. All the *S. aureus* strains were studied for the detection of antibiotic resistance genes (*blaZ*, *mecA*, *ermA*, *ermB*, *ermC*, *aacA-aphD*, and *tetM*) using the protocols of Gan et al. [27] and Deepak et al. [28].

The PCR amplification conditions were as follows: 5 min at 95 °C, 30 cycles of 30 s at 95 °C, annealing for 45 s at different temperatures for each gene (Supplementary Table S2), and extension for 45 s at 72 °C, followed by a final extension of 15 min at 72 °C.

Subsequently, the SCC*me*c Types I–V of MRSA were determined by PCR using the protocol described by Shah et al. [29]. The amplification conditions for the SCC*mec* I–V genes were as follows: 5 min at 94 °C, 30 cycles of 1 min at 94 °C, 1 min at 51 °C, and 1.5 min at 72 °C, followed by a final extension of 10 min at 72 °C (Supplementary Table S2). The products of the amplification were electrophoresed on 1% (*w*/*v*) agarose gel (UltraPure agarose, Invitrogen, Carlsbad, USA) using SYBR Green (Sigma-Aldrich, St. Louis, MO, USA). *S. aureus* ATCC 43300, *S. aureus* ATCC 33598, and *S. aureus* ATCC 6538 were used as the controls.

### 2.3. Antimicrobial Susceptibility Testing

Antimicrobial susceptibility was determined using the agar diffusion method according to the CLSI guidelines [30]. Suspensions of 0.5 McFarland of each *S. aureus* strain were cultured on Mueller–Hinton agar (MHA; Becton Dickinson Bioxon, Le Pont de Claix, France), incubated at 35 °C for 24 h. The following antimicrobial agents were included: penicillin (PE: 10 U); ciprofloxacin (CPF: 5 µg); clindamycin (CLM: 30 µg); erythromycin (E: 15 µg), tetracycline (TE: 30 µg); gentamicin (GE: 10 μg), and sulfamethoxazole–trimethoprim (SXT: 2.5/23.75 μg) (BBLTM Sensi-DiscTM, Becton Dickinson, Le Pont de Claix, France). After 24 h of incubation, the inhibition zone was measured and interpreted in accordance with the breakpoints for *S. aureus* [30]. Further, the minimum inhibitory concentration (MIC) of vancomycin (Sigma-Aldrich, St. Louis, MO, USA) and oxacillin (Sigma-Aldrich, St. Louis, MO, USA) was determined for all the isolates, following the broth microdilution method recommended by CLSI guidelines [30].

The criteria for defining multidrug resistance (MDR) in *S. aureus* are as follows [31] (one or more of these must apply): (i) an MRSA is always considered MDR; (ii) non-susceptibility to ≥1 agent in ≥3 antimicrobial categories [32]. In addition, the multiple antimicrobial resistance (MAR) index of each *S. aureus* was determined using the methods described by Krumperman [33] and Blasco et al. [34].

### 2.4. Disinfectant and Heavy Metal Sensitivity

Benzalkonium chloride (BC) (Sigma-Aldrich, St. Louis, MO, USA) was used to determine the sensitivity of the *S. aureus* strains using the protocol of Ebrahimi et al. [35] with modifications. The standard MIC was determined by the broth microdilution method. Briefly, 100 µL of bacterial suspension (~10^8^ CFU/mL) in Mueller–Hinton broth (MHB; Becton Dickinson Bioxon, Le Pont de Claix, France) was added to 100 µL BC at various concentrations of 100, 50, 25, 12.5, 6.2, 3.1, 1.5, and 0.7 µg/mL. Each strain was tested in triplicate, with the positive (100 µL of MHB + 100 µL of *S. aureus* ATCC 25923) and negative control wells containing only 200 µL of MHB. Cadmium chloride (CdCl_2_; Sigma-Aldrich, St. Louis, MO, USA) was used to determine the resistance of *S. aureus* to heavy metal cadmium. Each *S. aureus* isolate was adjusted to ~10^8^ CFU/mL and was inoculated onto the MHA supplemented with different concentrations of CdCl_2_ (400, 200, 100, 70, 50, 25, and 12.5 µg/mL), which were then incubated at 35 °C/24 h in triplicate. An isolate with growth on an agar plate was considered resistant/tolerant to the concentration of heavy metal [10,19].

### 2.5. Treatment with BC with or Without Milk Residues for Removal of Biofilm Methicillin-Resistant S. aureus (MRSA) and Methicillin-Susceptible S. aureus (MSSA)

Biofilms of MRSA and MSSA were developed on stainless steel (SS) coupons (AISI 316, 0.8 × 2.0 × 0.1 cm; CIMA Inoxidable, Jalisco, Mexico) using a protocol described by Avila-Novoa et al. [36]. The coupons with mono-species biofilms of MRSA and MSSA were treated with (i) BC in distilled H_2_O at 100 µg/mL or (ii) BC in distilled H_2_O at 100 µg/mL + 1% of milk residues (BC + MR). BC and BC + MR were applied at 22 °C with two exposure times: 5 and 10 min. After the exposure period, each coupon was transferred to Modified Letheen broth (MLB; Becton Dickinson Bioxon, Le Pont de Claix, France) for 30 min. Bacterial enumeration was estimated by standard plate counting on tryptic soy agar (TSA; Becton Dickinson Bioxon, Le Pont de Claix, France) at 35 °C for 24 h. Each assay was performed in triplicate, and the controls with distilled water and 1% milk residue were included.

### 2.6. Evaluation of Cell Viability and Scanning Electron Microscopy (SEM)

The cell viability of the MRSA and MSSA biofilms on the SS coupons was determined before and after the treatments mentioned in Section 2.5 using the LIVE/DEAD™ BacLight™ Bacterial Viability Kit (Thermo Fisher Scientific, Eugene, OR, USA) examined under a Nikon Eclipse E400 epifluorescence microscope. Simultaneously, SEM analysis of the SS coupons was performed (Section 2.5) according to the protocols described by Borucki et al. [37] and Fratesi et al. [38]. The biofilms were observed using a TESCAN Mira3 LMU scanning electron microscope (Tezcan, Czech Republic).

### 2.7. Statistical Analysis

Each experiment was carried out three times independently, and the data were subjected to analysis of variance (ANOVA) with post hoc comparisons using the least significant difference (LSD) test in Statgraphics Centurion XVI (StatPoint Technologies, Inc., Warrenton, VA, USA).

## 3. Results

### 3.1. Antimicrobial Resistance and Sensitivity to Disinfectants and Heavy Metals of MRSA and MSSA

A total of 41.7% (28/67; MIC of ≥4 μg/mL oxacillin) of the *S. aureus* strains harbored the *mecA* gene, indicating that they were MRSA; moreover, 20.8% (14/67) belonged to SCC*mec* Type II, 13.4% (9/67) to SCC*mec* Type V, and 7.4% (5/67) to SCC*mec* Type IV. In 58.2% (39/67) of the isolates, the *mecA* gene was not detected, and the isolate was considered MSSA.

Subsequently, all the isolates were screened for antibiotic resistance genes: erythromycin ribosome methylase *ermC* (2.9%; 2/67), *ermA* (2.9%; 2/67), *ermB* (10.4%; 7/67); and the bifunctional aminoglycoside N-acetyltransferase and aminoglycoside phosphotransferase with *aacA-aphD* (10.4%; 7/67), which showed resistance to gentamicin. Tetracycline (*tetM*) and penicillin resistance (*blaZ*) were detected in 17.9% (12/67) and 88% (59/67), respectively (Table 1).

Additionally, the antimicrobial sensitivity and resistance profiles of the MRSA and MSSA isolates against the 8 antibiotics tested are shown in Table 1. Overall, the *S. aureus* isolates (both MRSA and MSSA) showed susceptibility to trimethoprim–sulfamethoxazole (82%; 55/67), tetracycline (83.5; 56/67), erythromycin (86.5%; 58/67), gentamicin (88%; 59/67), and vancomycin (92.5%; 62/67).

Table 2 shows the genetic characteristics and the antibiotic resistance profiles of the MRSA isolates.

In addition, 71.64% (48/67) of MRSA and MSSA were MDR (Table 3), and nineteen *S. aureus* isolates were not considered to be MDR (Table 3). The MAR index for MRSA and MSSA isolates ranged from 0.12 to 0.75, with 34.3% presenting an MAR index of 0.25. In addition, the MICs of 97% (65/67) of isolates lay between 1.56 and 25 µg/mL BC, while 2.9% (2/67) had an MIC of 50 µg/mL.

Overall, 100% of *S. aureus* isolates were cadmium-resistant; however, 62.6% (42/67) were resistant to cadmium at >400 µg/mL CdCl_2_ [MRSA (34.3%; 23/67) and MSSA (28.3%; 19/67)], and 38.5% (25/67) were resistant to cadmium at 25–200 µg/mL of CdCl_2_ (Table 4).

### 3.2. Reduction in Biofilms of MSSA and MRSA with BC with or Without Milk Residues

**Figure 1:**
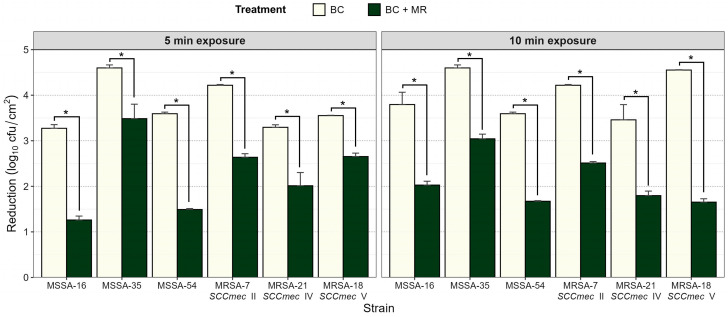
Effect of BC alone and in the presence of milk residues (MRs) on biofilm reduction in MSSA and MRSA strains. Biofilm reduction was assessed after 5 and 10 min of exposure. Data represent mean ± SD of three independent experiments. Asterisks (*) indicate statistically significant differences between treatments (*p* < 0.05).

Six *S. aureus* strains (MSRA-7, MSRA-18, MSRA-21, MSSA-16, MSSA-35, and MSSA-54) were selected based on their genotypic and phenotypic characteristics associated with SCC*mec* and antimicrobial resistance (Table 2). In addition to the ability to produce biofilm, it is essential to highlight that these strains have already been characterized as biofilm formers by detecting the responsible genes in their genomes [21,25]. Subsequently, it was determined that there was a significant difference in the treatments with BC (100 µg/mL) and BC + MR (100 µg/mL+ 1% milk residue*s*), which was applied at 22 °C (*p* < 0.05). The treatment with BC + MR led to a smaller reduction in biofilm (2.11–2.25 log_10_ cfu/cm^2^; *p* < 0.05) with exposure times of 5 and 10 min compared to BC (3.75–4.03 log_10_ cfu/cm^2^ at 5–10 min) (Figure 1).

MSSA-35 had a larger biofilm reduction (4.59 ± 0.06 log_10_ cfu/cm^2^; *p* < 0.05 at 5 min) with the BC treatment compared to MRSA-7 SCC*mec* II (4.22 ± 0.01 log_10_ cfu/cm^2^), MSSA-54 (3.59 ± 0.03 log_10_ cfu/cm^2^), MRSA-18 SCC*mec* V (3.55 ± 0.01 log_10_ cfu/cm^2^), MRSA-21 SCC*mec* IV (3.30 ± 0.05 log_10_ cfu/cm^2^), and MSSA-16 (3.27 ± 0.08 log_10_ cfu/cm^2^).

However, in the 10 min exposure BC treatment, there was a greater reduction in biofilm (4.22–4.59 log_10_ cfu/cm^2^; *p* < 0.05) formed by the strains MRSA-7 SCC*mec* II, MSSA-35, and MRSA-18 SCC*mec* V compared to MSSA-16, MRSA-21 SCC*mec* IV, and MSSA-54 (3.46–3.79 log_10_ cfu/cm^2^).

In addition, MSSA-16 and MSSA-54 (1.26–1.49 log_10_ cfu/cm^2^; *p* < 0.05) exhibited a smaller reduction in biofilm in the BC + MR treatment with an exposure time of 5 min compared to MRSA-21 SCC*mec* IV, MRSA-7 SCC*mec* II, MSSA-35, and MRSA-18 SCC*mec* V. MSSA-35 had a biofilm reduction of 3 log_10_ cfu/cm^2^ (*p* < 0.05) at 10 min exposure time with BC + MR compared to MSSA-16, MRSA-7 SCC*mec* II, MRSA-21 SCC*mec* IV, MRSA-18 SCC*mec* V, and MSSA-54.

**Figure 2:**
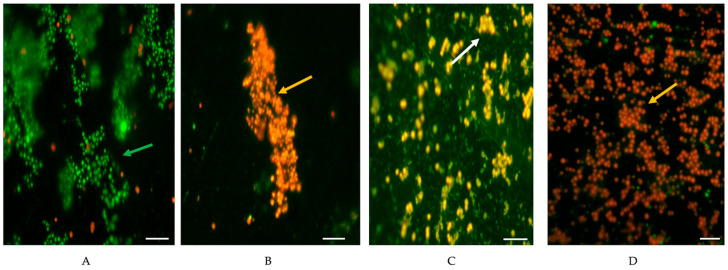
Micrographs of biofilms before and after removal treatments. Biofilms of MRSA and MSSA were developed on SS during 192 h of incubation at 35 °C. Micrographs were obtained by epifluorescence microscopy (100×), stained with LIVE/DEAD™ BacLight™ before and after removal treatments. (**A**) MRSA-7 SCC*mec* II before applied treatment; (**B**) MRSA-7 SCC*mec* II after applied treatment with BC (100 µg/mL, 5 min, 22 °C); (**C**) MSSA-54 after applied treatment with BC + MR (100 µg/mL + 1% of milk residues, 10 min, 22 °C); (**D**) MRSA-18 SCC*mec* V after applied treatment with BC (100 µg/mL, 10 min, 22 °C). White bar scale indicates 5 μm. Distinctive fluorescence and patterns can be linked to different cellular states. As for LIVE/DEAD™ BacLight™ stain, SYTO9 penetrates all bacterial membranes and stains cells green (green arrows). Propidium iodide only penetrates cells with damaged membranes. Fluorescing cells turn red (orange arrows), and bacterial cells in an intermediate state between alive and dead or damaged are stained yellow-green (white arrows).

Figure 2 shows the live and dead cells of the MRSA and MSSA biofilms before and after the treatment with BC and BC + MR.

**Figure 3:**
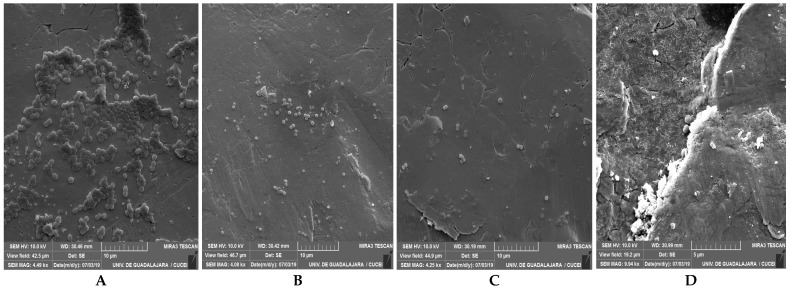
Micrographs of biofilms before and after removal treatments. Biofilms of MRSA and MSSA were developed on SS during 192 h of incubation at 35 °C. Micrographs were obtained by scanning electron microscopy (SEM) before and after removal treatments. (**A**) MRSA-7 SCC*mec* II before applied treatment; (**B**) MRSA-7 SCC*mec* II after applied treatment with BC (100 µg/mL, 5 min, 22 °C); (**C**) MSSA-35 after applied treatment with BC (100 µg/mL, 10 min, 22 °C); (**D**) MRSA-18 SCC*mec* V after applied treatment with BC (100 µg/mL + 1% of milk residues, 10 min, 22 °C).

SEM analysis of the biofilms formed by the representative mono-species MRSA and MSSA revealed that the cells were linked to each other and embedded in dense EPS (Figure 3).

## 4. Discussion

*S. aureus* and MRSA are considered to be a public health problem due to their ability to contaminate various foods, such as milk and dairy products, as well as colonize and infect humans and animals. In 2024, MRSA was ranked 14th on the Bacterial Priority Pathogens List (BPPL). They are considered high-priority-group pathogens and are therefore a global health problem [12].

In this study, we analyzed a total of 67 *S. aureus* samples collected from food contact surfaces in the dairy industry, 41.7% of which were MRSA. Some authors have reported a similar prevalence for MRSA (51.6–95%) in raw milk samples (including cow, clinical mastitis, camel, and horse milk) [39,40]; however, several authors identified a lower prevalence (1.23–14%) for MRSA collected from sheep and goat bulk tanks, animal (rectal and nasal swabs) mastitis milk samples, dairy products, and ready-to-eat (RTE) foods [31,36,41,42,43,44]. Likewise, Kotzamanidis et al. [5] did not detect MRSA among the *S. aureus* strains recovered from goats, sheep, and bovines with clinical and subclinical mastitis. This investigation suggests that the prevalence of *S. aureus* and MRSA is associated with various factors, including (i) a diversity of sources and mechanisms of contamination during the obtaining of milk and the finished product, where food contact surfaces and food handlers are involved [20,21,45]; (ii) the sample size, type of food, and methods used for the detection and characterization of pathogens; (iii) geographical distribution of the prevalence of *S. aureus* in developed or developing countries [31]; and (iv) implementation and verification of control measures within the industry, such as sanitary prerequisites, good agricultural practices, and standard operating procedures for sanitation.

Regarding genetic characterization, SCC*mec* Types II (20.8%), V (13.4%), and IV (7.4%) were detected in this study. Similarly, other studies have reported SCC*mec* Types II (28.4–94%), IV (6–22.2%), and V (0.9%) in mastitis milk (clinical and subclinical cow), clinical strains of MRSA, clinical samples, and surgical materials [42,46,47]. Likewise, Annamanedi et al. [6] reported that the prevalence of SCC*mec* IVb, SCC*mec* IVd, and SCC*mec* V was 16.2%, 1.8% and 1.2% among the clinical and subclinical mastitis cases. SCC*mec* IVa has been associated with serious human infections and is spread primarily in community settings in humans and livestock populations [31,48,49]. Notably, the characterization of the pathogens and risk associated with food categories would aid in the prevention and treatment of infections with these pathogens in humans and animals.

Overall, the MSSA and MRSA isolates detected in this study exhibited antibiotic resistance (Table 1). Previous investigations revealed similar wide-ranging prevalences of antimicrobial resistance to penicillin (58.5–92.5%), ciprofloxacin (4.6–22.2%), clindamycin (17.5–97.5), erythromycin (8.3–87.5%), vancomycin (3.2%), tetracycline (11.3–77.8%), gentamicin (12.3–30.6%), and sulfamethoxazole–trimethoprim (19.4%) in *S. aureus* and MRSA isolates from raw milk samples of different varieties (goat bulk tank milk, mastitis milk samples, dairy products, and ready-to-eat (RTE) foods) [31,39,40,42,50,51]. The use of antibiotics in animal production, overprescription, and inappropriate use of antimicrobials in animal and human medicine may have contributed to the prevalence of heterogeneity of resistance to various antimicrobial classes [36,51,52].

Additionally, 71.64% of the MRSA (41.79%; 28/67) and MSSA (29.85%; 20/67) strains was MDR (Table 3), with an MAR index of >0.25 (86.56%; ranged 0.25–0.75), indicating a higher risk of contamination (MAR index of >0.2) associated with the continuous use of antibiotics [33]. Other investigators have also reported similar observations [10,19,40] that demonstrated the prevalence of MDR *S. aureus* (9.4–90.8%) with an MAR index of >0.2; however, the variation in the prevalence of MDR and MAR indexes is associated with the geographical area, seasons, sample size [53], the amount of antimicrobial classes, and the phenotypic and genotypic characteristics of *S. aureus*.

The detection of *mec*A, *bla*Z, *tet*M, *aac*A-*aph*D, *erm*A, *erm*B, and *erm*C in the *S. aureus* isolates in our study may be related to various resistance mechanisms, such as penicillin-binding protein 2a (PBP2a) activity and hyperproduction of β-lactamases, which confers resistance to β-lactam antibiotics [9,31], and the efflux pump and the bifunctional enzyme with acetyltransferase and phosphotransferase activity that confers resistance to tetracycline and gentamicin and methylases that modify A20258/A2059 in rRNA 23S and inhibit macrolide, lycosamide, and streptogramin B MLSB [54]. This is in accordance with the detection of *mec*A, *bla*Z, *tet*M, *aac*A-*aph*D, *erm*A, *erm*B, and *erm*C in *S. aureus* and MRSA isolates from milk samples and clinical specimens [28,39,40,55].

This research suggests that there is a high prevalence of resistance to β-lactams used in the treatment of bovine mastitis, such as penicillin and ampicillin [56,57], indicating the necessity for regulatory adjustments and optimized treatment protocols to strengthen control and regulation of bovine mastitis [40,42]. Furthermore, resistance to MLS_B_, which is a second-line drug used by patients with an allergy to β-lactams [43], can prolong and worsen the disease, increase healthcare costs, and increase the risk of death [58].

Additionally, the presence of heavy metal residues and antibacterial biocides can co-select antibiotic-resistant bacteria (cross-resistance) or cause co-resistance via interaction and genetic exchange through plasmids [18]. Resistance to cadmium in *S. aureus* is caused by the gene *cadA* that encodes efflux mechanisms consisting of Zn(II)/Cd(II)/Pb(II)-translocating ATPase [59].

In this study, 62.6% of the *S. aureus* isolates were cadmium-resistant (≥400 µg/mL). Other researchers [10,19] have reported similar findings regarding the progressive cadmium resistance (from 50 to 1500 µg/mL) [56.3% and 91.2% (100 µg/mL) or 39.4% and 88.9% (1500 µg/mL)] in *S. aureus* collected from livestock animals and the environment. Environmental cadmium contamination in different ecological settings, including agricultural soil and ground-, drinking, and wastewater, can be attributed to anthropogenic sources, including the industrial sector, such as the use of phosphate fertilizers and detergents within the sanitary or agricultural prerequisites [60,61,62]. Notably, *S. aureus* exhibits co-expression of antibiotic and heavy metal resistance, which complicates the prevention or treatment of pathologies caused by *S. aureus* and MRSA in the host [19,61] or in disinfection processes for the control of foodborne pathogens in the food industry.

Quaternary ammonium compounds (QACs), such as BC, are used in the food industry as sanitizers to control foodborne pathogens [63]. In this study, 97% of *S. aureus* showed MICs = 1.56 ≤ 25 µg/mL and had a 2.9% MIC = 50 µg/mL (Table 4); moreover, the BC [4MIC (MIC = 6.25 µg/mL)] more effectively removed the MRSA-7 SCC*mec* II, MRSA-18 SCC*mec* V, and MSSA-35 strain biofilms (*p* < 0.05) than the MSSA-16, MRSA-21 SCC*mec* IV and, MSSA-54 types (Figure 1). Therefore, BC is an effective disinfectant for preventing environmental niches and forming a biofilm in industry to control MRSA and MSSA on food contact surfaces. Indeed, other studies have revealed the antibacterial activity of BC against *S. aureus* and MRSA at conventional in-use concentrations for sanitizers [63,64,65]. In fact, the MIC or a serial increase in this (from 2MIC to 8MIC) for BC inhibited planktonic growth and biofilm formation with *S. aureus* and *S. epidermidis* CIP54124 [35,66].

The co-occurrence of heavy metal and antibiotic resistance is highly relevant, as the former can enhance the latter or lead to cross-resistance between these compounds, as previously reported [67]. Komijani et al. [68] demonstrated a strong correlation between the presence of antimicrobial resistance genes and heavy metal concentrations in aquatic environments, an association even stronger than that observed with the antibiotics themselves. Similarly, Zhou et al. [69] found that in dairy farm environments, ARGs and metal resistance genes are positively correlated, suggesting that heavy metals not only promote the emergence of metal resistance but also contribute to the co-selection of antibiotic resistance. This phenomenon is particularly significant in dairy facilities, where MRSA and MSSA strains are prevalent.

Finally, the anti-biofilm effect of MRSA and MSSA of BC is affected by the presence of organic matter (*p* < 0.05); in addition, MRSA-18 SCC*mec* V, MSSA-54, and MRSA-7 SCC*mec* II showed the lowest biofilm reduction in BC + MR with 10 min of exposure (*p* < 0.05) compared to MSSA-16, MRSA-21 SCC*mec* IV, and MSSA-35 (Figure 1) in this study. This result suggests that the decrease in BC efficiency is related to (i) the BC resistance mechanism or *S. aureus* biofilm matrix components decreasing the diffusion of sanitizers or antibiotics [39,66], (ii) sub-MIC exposure to BC inducing biofilm formation of *S. aureus* [35], (iii) the decrease in biofilm shrinkage associated with the slow growth rate of *S. aureus* embedded within the biofilm matrix [70], and (iv) the presence of organic matter favors pre-conditioning on the surface and bacterial adhesion or the low pH limiting the production of extracellular proteases within the biofilm matrix, favoring its formation [71,72,73]. Consequently, an *S. aureus* biofilm is a potential source of contamination among food products, generating major food safety problems and economic losses for the food industry. In addition, hazard characterization based on the determination of antimicrobial resistance factors or co-resistance to heavy metals and disinfectants is helpful for constant improvement of treatments and cleaning and disinfection procedures used in the food industry.

## 5. Conclusions

This study demonstrated that *S. aureus* strains isolated from FCSs in the dairy industry exhibit a high prevalence of methicillin and multidrug resistance, with SCC*mec* Types II, IV, and V being the most common. The co-occurrence of resistance to antibiotics, benzalkonium chloride, and cadmium highlights the capacity of these strains to persist in industrial environments, even under disinfection conditions. These findings underscore the urgent need to strengthen hygiene monitoring and antimicrobial stewardship within food production facilities. More importantly, this work provides molecular and phenotypic evidence that food contact surfaces can act as long-term reservoirs for MRSA and MSSA, representing a critical point for intervention to reduce the risk of foodborne transmission and improve food safety.

## Figures and Tables

**Table 1:** Antimicrobial susceptibility test results for MRSA and MSSA.

Antimicrobial Class According to the WHO		Antibiotic	No. (%) of MRSA (n = 28) and MSSA (n = 39) Strains					
			Resistant		Susceptible		Intermediate	
			MRSA	MSSA	MRSA	MSSA	MRSA	MSSA
Highly important	Lincosamides	CLM	19 (28.3)	25 (37.3)	9 (13.4)	14 (20.8)	-	-
	Sulfonamides	STX	5 (7.4)	7 (10.4)	23 (34.3)	32 (47.7)	-	-
	Cyclic peptides	TE	8 (11.9)	3 (4.4)	20 (29.8)	36 (53.7)	-	-
Critically important	Macrolides	E	5 (7.4)	4 (5.9)	23 (34.3)	35 (52.2)	-	-
	Aminoglycosides	GE	6 (8.9)	2 (2.9)	22 (32.8)	37 (55.2)	-	-
	Fluoroquinolones	CPF	11 (11.9)	22 (32.8)	12 (17.9)	10 (14.9)	5 (7.4)	7 (10.4)
	β-Lactams	PE	28 (41.7)	39 (58.2)	-	-	-	-
	Glycopeptides	VA *	-	5 (7.4)	28 (41.7)	34 (50.7)	-	-

Abbreviations for antibiotics were defined in Section 2.3. * MIC Test of vancomycin by CLSI guidelines.

**Table 2:** Genetic analysis and antibiotic resistance results of MRSA (n = 28) isolated from food contact surfaces.

Strain No.	SCC*mec*	Antimicrobial Resistance Genes						Antibiotic Resistance	MAR Index
		ermB	ermC	ermA	tetM	blaZ	aacA-aphD		
MSRA-2	SCC*mec* II	-	-	-	-	+	+	PE + GE	0.25
MSRA-6	SCC*mec* II	-	-	-	-	+	-	PE + CLM	0.25
MSRA-7	SCC*mec* II	+	+	-	+	+	-	PE + CPF + CLM + E + TE	0.62
MSRA-8	SCC*mec* II	-	-	-	+	+	-	PE + CLM + TE	0.37
MSRA-9	SCC*mec* II	-	-	-	-	+	-	PE + CLM	0.25
MSRA-10	SCC*mec* II	-	-	-	-	+	-	PE	0.12
MSRA-11	SCC*mec* II	-	-	-	-	+	-	PE	0.12
MSRA-23	SCC*mec* II	+	-	-	+	+	+	PE + CPF + CLM + E + TE + GE	0.75
MSRA-27	SCC*mec* II	+	-	-	+	+	-	PE + CPF + CLM + E + TE + GE	0.75
MSRA-28	SCC*mec* II	-	-	-	-	+	-	PE	0.12
MSRA-37	SCC*mec* II	-	-	-	+	+	-	PE + CLM + TE	0.37
MSRA-40	SCC*mec* II	-	-	-	-	-	-	PE + CLM	0.25
MSRA-43	SCC*mec* II	-	-	-	+	+	-	PE + CLM + TE + STX	0.50
MSRA-44	SCC*mec* II	-	-	-	-	+	-	PE + CLM	0.25
MSRA-3	SCC*mec* IV	-	-	+	-	+	+	PE + CPF + CLM + E + GE	0.62
MSRA-13	SCC*mec* IV	+	-	-	-	-	+	PE + CLM + E + GE	0.50
MSRA-17	SCC*mec* IV	-	-	-	-	+	-	PE + CPF + CLM	0.37
MSRA-21	SCC*mec* IV	-	-	-	+	+	-	PE + CPF + CLM + TE + STX	0.62
MSRA-56	SCC*mec* IV	-	-	-		+	-	PE	0.12
MSRA-4	SCC*mec* V	-	-	-	+	+	-	PE + TE	0.25
MSRA-5	SCC*mec* V	-	-	-	-	+	-	PE + CPF + CLM + STX	0.50
MSRA-12	SCC*mec* V	-	-	-	+	+	-	PE + CPF + CLM + TE + STX	0.62
MSRA-15	SCC*mec* V	-	-	-	-	+	-	PE + CLM + STX	0.37
MSRA-18	SCC*mec* V	-	-	-	-	-	-	PE + CPF	0.25
MSRA-24	SCC*mec* V	-	-	-	-	+	-	PE	0.12
MSRA-34	SCC*mec* V	-	-	-	-	+	+	PE + GE	0.25
MSRA-39	SCC*mec* V	-	+	-	-	+	-	PE + CPF + CLM + E	0.50
MSRA-62	SCC*mec* V	-	-	-	-	-	-	PE + CPF + CLM	0.37

SCC*mec*, staphylococcal chromosomal cassette *mec*. Abbreviations for antibiotics were defined in Section 2.3.

**Table 3:** Antimicrobial resistance patterns and multiple antimicrobial resistance indices of *S. aureus* (n = 67) from food contact surfaces.

No. of Antimicrobial	Resistance Profile	*Staphylococcus aureus*		MAR Index
		No. (%) of MSSA (n = 39)	No. (%) of MRSA (n = 28)	
6	PE + CPF + CLM + E + TE + GE	-	2 (2.98)	0.75
	PE + CPF + CLM + VA + GE + STX	1 (1.49)	-	
	PE + CPF + CLM + E + TE + STX	1 (1.49)	-	
5	PE + CPF + CLM + TE + STX	-	2 (2.98)	0.62
	PE + CPF + CLM + E + TE	-	1 (1.49)	
	PE + CPF + CLM + E + GE	-	1 (1.49)	
4	PE + CLM + TE + STX	-	1 (1.49)	0.50
	PE + CLM + E + GE	-	1 (1.49)	
	PE + CPF + CLM + STX	-	1 (1.49)	
	PE + CPF + CLM + E	-	1 (1.49)	
	PE + CPF + CLM + STX	3 (4.47)	-	
	PE + CPF + CLM + VA	1 (1.49)	-	
	PE + CPF + CLM + TE	1 (1.49)	-	
	PE + CLM + E + VA	1 (1.49)	-	
	PE + CLM + VA + STX	1 (1.49)	-	
3	PE + CPF + CLM	9 (13.43)	2 (2.98)	0.37
	PE + CLM + TE	-	2 (2.98)	
	PE + CLM + STX	-	1 (1.49)	
	PE + E + TE	1 (1.49)	-	
	PE + CLM + VA	1 (1.49)	-	
2	PE + TE	-	1 (1.49)	0.25
	PE + GE	1 (1.49) *	2 (2.98)	
	PE + CPF	6 (8.95) *	1 (1.49)	
	PE + CLM	6 (8.95) *	4 (5.97)	
	PE + STX	1 (1.49) *	-	
	PE + E	1 (1.49) *	-	
1	PE	1 (1.49) *	3 (4.47)	0.12
	PE	2 (2.98) *	2 (2.98)	
	PE	1 (1.49) *	-	

* *S. aureus* is not MDR. Abbreviations for antibiotics were defined in Section 2.3.

**Table 4:** MIC of BC and resistance to CdCl_2_ in MRSA and MSSA.

	µg/mL	No. (%) of MRSA (n = 28)	No. (%) of MSSA (n = 39)	Total
BC (MIC)	50	2 (2.9)	-	2 (2.9)
	25	8 (11.9)	12 (17.9)	20 (29.8)
	12.5	2 (2.9)	2 (2.9)	4 (5.9)
	6.25	6 (8.9)	1 (1.4)	7 (10.4)
	3.12	2 (2.9)	7 (10.4)	9 (13.4)
	1.56	8 (11.9)	17 (25.3)	25 (37.3)
ClCd_2_	Higher 400	23 (34.3)	19 (28.3)	42 (62.6)
	200	-	1 (1.4)	1 (1.4)
	100	-	1 (1.4)	1 (1.4)
	70	1 (1.4)	7 (10.4)	8 (11.9)
	50	2 (2.9)	3 (4.4)	5 (7.4)
	25	2 (2.9)	8 (11.9)	10 (14.9)

## Data Availability

The original contributions presented in this study are included in the article/Supplementary Materials. Further inquiries can be directed to the corresponding authors.

## Supplementary Materials

The following supporting information can be downloaded at https://www.mdpi.com/article/10.3390/life15121811/s1, Table S1. Distribution and genetic analysis of *S. aureus* (n = 67) isolates collected from food contact surfaces (FCSs) in the dairy industry; Table S2: Primers for amplification of resistance genes in *S. aureus* and the conditions of annealing for PCR.
