# Supplementary material for: Genetic Profiling of MRSA and MSSA from Food Contact Surfaces: Antibiotic, Heavy Metal and Benzalkonium Chloride Resistance
Source: Life (Basel). 2025 Nov 26;15(12):1811. doi: 10.3390/life15121811 (PMC12734234; doi:10.3390/life15121811)
Supplement: Supplementary file 1 [file life-15-01811-s001.zip › Supplementary Table S1.pdf]

Supplementary Table S1. Distribution and genetic analysis of *S. aureus* (n = 67) isolates collected from food contact surfaces (FCSs) in the dairy industry.

Isolate ID	Date of collection	Source	Region of Jalisco State	Enterotoxin genes	Phenotypic and genotypic characteristics associated with *S. aureus* biofilm formation		
					**Genotypes biofilm related**	**Polystyrene Microtiter Plates Biofilm Formation**	**Biofilm phenotype on CRA**
SA-1	2018	FCS-PP	Eastern	----	*clfA+clfB+bap+agrD+sigB+icaAD*	MB	BP
SA-2	2018	FCS-SS	Eastern	----	*agrD+sigB+ icaADBC*	MB	BP
SA-3	2018	FCS-PP	Eastern	----	*agrD+sigB+ icaADBC*	MB	BP
SA-4	2018	FCS-SS	Northern	*sec + sed + seg + sej*	*clfA+clfB+fnbpA+fnbpB+bap+agrD+sigB+sar+icaADBC*	MB	BP
SA-5	2018	FCS-SS	Eastern	----	*clfA+clfB+icaADBC*	MB	BP
SA-6	2018	FCS-PP	Eastern	----	*clfA+clfB+agrD+sigB+icaADBC*	MB	BP
SA-7	2018	FCS-SS	Eastern	*sej*	*clfA+clfB+icaADBC*	MB	BP
SA-8	2018	FCS-PP	Eastern	----	*clfA+clfB+sigB+icaADBC*	MB	BP
SA-9	2018	FCS-SS	Northern	*sec + sed + seh + sej*	*clfA+clfB+fnbpA+fnbpB+bap+agrD+sigB+sar+icaADBC*	MB	BP
SA-10	2018	FCS-SS	Eastern	----	*clfA+clfB+fnbpA+fnbpB+agrD+sar+icaADBC*	MB	BP
SA-11	2018	FCS-SS	Eastern	----	*agrD+sigB+icaADBC*	MB	BP
SA-12	2018	FCS-SS	Eastern	----	*clfA+clfB+icaADBC*	MB	BP
SA-13	2018	FCS-SS	Northern	*seb + sed + sei*	*agrD+sigB+icaADBC*	SB	BP
SA-14	2018	FCS-SS	Eastern	----	*sigB+sar-bap+icaAD*	MB	BP
SA-15	2018	FCS-SS	Eastern	----	*clfA+clfB+icaADBC*	MB	BP
SA-16	2018	FCS-SS	Eastern	*sed + seg*	*agrD+sigB+icaADBC*	MB	BP
SA-17	2018	FCS-SS	Eastern	----	*fnbpA+fnbB-sigB+icaADBC*	MB	BP
SA-18	2018	FCS-SS	Northern	*sec + sej*	*ClfB+clfB+sigB+icaADBC*	SB	BP
SA-19	2018	FCS-SS	Northern	*sea + sej*	*agrD+sigB+icaADBC*	MB	BP
SA-20	2018	FCS-SS	Eastern	----	*icaAD*	MB	BP
SA-21	2018	FCS-SS	Northern	*sei + sej*	*agrD+sigB+sar+bap+icaADBC*	MB	BP
SA-22	2018	FCS-SS	Eastern	*sej*	*agrD+sigB+sar+icaAD*	MB	BP
SA-23	2018	FCS-SS	Eastern	*sed + seg*	*agrD+sigB+icaADBC*	MB	BP
SA-24	2018	FCS-PP	Eastern	----	*agrD+sigB+sar+icaADBC*	MB	BP
SA-25	2018	FCS-PP	Eastern	----	*clfA+clfB+sigB+icaD*	MB	NCF
SA-26	2018	FCS-PP	Eastern	----	*clfA+clfB+fnbpA+fnbpB+sigB*	MB	NCF
SA-27	2018	FCS-SS	Central-Eastern	----	*clfA+clfB+icaADBC*	MB	BP
SA-28	2018	FCS-SS	Central-Eastern	----	*agrD+sigB+sar+icaADBC*	MB	BP
SA-29	2018	FCS-PP	Eastern	*sej*	*clfA+clfB+icaBC*	MB	BP
SA-30	2018	FCS-PP	Eastern	----	*clfA+clfB+agrD+icaAD*	MB	BP
SA-31	2018	FCS-PP	Eastern	----	*clfA+clfB+agrD+icaADBC*	MB	BP
SA-32	2018	FCS-PP	Eastern	----	*agrD+sigB+sar+icaAD*	MB	BP
SA-33	2018	FCS-PP	Eastern	----	*clfA+clfB+fnbpA+fnbpB+agrD+icaAD*	SB	BP
SA-34	2018	FCS-SS	Eastern	----	*agrD+sigB+icaADBC*	MB	BP

SA-35	2018	FCS-PP	Northern	*sed + seg + sej*	*agrD+sigB+sar+icaADBC*	SB	BP
SA-36	2018	FCS-PP	Eastern	----	*clfA+clfB+agrD+sigB+icaAD*	MB	BP
SA-37	2018	FCS-SS	Central-Eastern	*sej*	*agrD+sigB+sar+icaADBC*	MB	BP
SA-38	2018	FCS-SS	Northern	----	*sigB+sar+icaB*	MB	BP
SA-39	2018	FCS-SS	Eastern	----	*clfA+clfB+agrD+sigB+sar+icaADBC*	MB	BP
SA-40	2018	FCS-PP	Central-Eastern	----	*fnbpA+fnbpB+agrD+sigB+sar+icaADBC*	SB	BP
SA-41	2018	FCS-PP	Eastern	----	*fnbpA+fnbB-agrD*	MB	NCF
SA-42	2018	FCS-SS	Northern	----	*clfA+clfB+sar+icaAD*	MB	BP
SA-43	2018	FCS-PP	Central-Eastern	----	*fnbpA+fnbpB+agrD+sigB+sar+icaADBC*	MB	BP
SA-44	2018	FCS-PP	Central-Eastern	----	*fnbpA+fnbpB+agrD+sigB+sar+icaADBC*	MB	BP
SA-45	2018	FCS-SS	Northern	*sej*	*clfA+clfB+fnbpA+fnbpB+bap+agrD+sigB+sar+icaAD*	MB	BP
SA-46	2018	FCS-PP	Eastern	----	*clfA+clfB+fnbpA+fnbpB+bap+icaD*	MB	BP
SA-47	2018	FCS-SS	Northern	*sed*	*sigB+sar+icaAD*	MB	BP
SA-48	2018	FCS-PP	Eastern	*sei*	*agrD+sigB+sar+icaAD*	MB	BP
SA-49	2018	FCS-PP	Eastern	*sed + seg + sej*	*sigB+sar+icaADBC*	MB	BP
SA-50	2018	FCS-SS	Central-Eastern	*sej*	*clfA+clfB+fnbpA+fnbpB+bap+agrD+sigB+sar+icaADBC*	MB	BP
SA-51	2018	FCS-PP	Northern	*sed + seg + sei*	*agrD+sigB+sar+icaADBC*	MB	BP
SA-52	2018	FCS-SS	Eastern	*sej*	*sigB+sar+icaAD*	MB	BP
SA-53	2018	FCS-SS	Northern	*sei + sej*	*clfA+clfB+sigB+icaADBC*	MB	BP
SA-54	2018	FCS-SS	Northern	*seh*	*sigB+sar+icaADBC*	SB	BP
SA-55	2018	FCS-SS	Eastern	----	*agrD+sigB+sar+icaADBC*	MB	BP
SA-56	2018	FCS-PP	Eastern	----	*sigB+sar+icaADBC*	MB	BP
SA-57	2018	FCS-SS	Northern	*sed*	*agrD+sigB+sar+icaADBC*	MB	BP
SA-58	2018	FCS-PP	Eastern	----	*clfA+clfB+agrD+sigB+icaAD*	MB	BP
SA-59	2018	FCS-SS	Northern	*seh + sej*	*clfA+clfB+fnbpA+fnbpB+sigB+sar+icaAD*	MB	BP
SA-60	2018	FCS-SS	Northern	*sed*	*fnbpA+fnbpB+agrD+sigB+sar+icaADBC*	MB	BP
SA-61	2018	FCS-SS	Northern	*sej*	*clfA+clfB+agrD+sigB+icaADBC*	MB	BP
SA-62	2018	FCS-PP	Central-Eastern	----	*clfA+clfB+agrD+sigB*	MB	NCF
SA-63	2018	FCS-SS	Northern	*sej*	*agrD+sigB+sar+icaADBC*	MB	BP
SA-64	2018	FCS-PP	Northern	*sed*	*clfA+clfB+agrD+sigB+icaAD*	MB	BP
SA-65	2018	FCS-SS	Northern	*sej*	*agrD+sigB+sar+icaADBC*	MB	BP
SA-66	2018	FCS-SS	Northern	*sej*	*clfA+clfB+fnbpA+fnbpB+agrD+sigB+sar+icaADBC*	MB	BP
SA-67	2018	FCS-SS	Central-Eastern		*clfA+clfB+fnbpA+fnbpB+sar+icaADBC*	MB	BP

SA, *S. aureus*; FCS, food contact surfaces; FCS-PP, FCS-polypropylene; FCS-SS, FCS-stainless steel; CRA, Congo red agar plates; NCF, Noncharacteristic phenotype in CRA (colonies pink with dark centers resembling bull's eyes); MB, MB, SB, SB; BP, Biofilm producers in CRA (crusty black colonies, with a dry filamentous appearance). References [21,25-26].
