# Supplementary material for: Genetic Profiling of MRSA and MSSA from Food Contact Surfaces: Antibiotic, Heavy Metal and Benzalkonium Chloride Resistance
Source: Life (Basel). 2025 Nov 26;15(12):1811. doi: 10.3390/life15121811 (PMC12734234; doi:10.3390/life15121811)
Supplement: Supplementary file 1 [file life-15-01811-s001.zip › Supplementary Table S2.pdf]

Supplementary Table S2. Primers for amplification of resistance genes in *S. aureus* and the conditions of annealing for PCR.

Gen	**Size (bp)**	**Sequence (5′—3′)**	**Annealing temperatura (ºC)**	**Reference**
blaZ	314	F-AACACCTGCTGCTTTCGGTA<br>R-CACTCTTGGCGGTTTCACTT	55.5	[27]
ermA	311	F-CTACACTTGGCTTAGGATGA<br>R-AGTGACTAAAGAAGCGGTAA	56.5	[27]
ermB	414	F-TAACGACGAAACTGGCTAA<br>R-CTGTGGTATGGCGGGTAA	56	[27]
ermC	375	F-GAGGCTCATAGACGAAGAAA<br>R-AAGTTCCCAAATTCGAGTAA	54.5	[27]
aacA-aphD	178	F-ATTGAAGATTTGCCAGAACA<br>R-CACTATCATAACCACTACCG	56.5	[27]
tetM	406	F-GTGGACAAAGGTACAACGAG<br>R-CGGTAAAGTTCGTCACACAC	62	[28]
mecA	310	F-GTAGAAATGACTGAACGTCCGATAA<br>R-CCAATTCCACATTGTTTCGGTCTAA	50	[28]
SCC*mec* I	613	F-GCTTTAAAGAGTGTCGTTACAGG<br>R-GTTCTCTCATAGTATGACGTCC	51	[29]
SCC*mec* II	398	F-CGTTGAAGATGATGAAGCG<br>R-CGAAATCAATGGTTAATGGACC	51	[29]
SCC*mec* III	280	F-CCATATTGTGTACGATGCG<br>R-CCTTAGTTGTCGTAACAGATCG	51	[29]
SCC*mec* IV	776	F-GCCTTATTCGAAGAAACCG<br>R-CTACTCTTCTGAAAAGCGTCG	51	[29]
SCC*mec* V	325	F-GAACATTGTTACTTAAATGAGCG<br>R-TGAAAGTTGTACCCTTGACACC	51	[29]

F, Forward primer; R, reverse
